# 2-(4-Fluoro­phen­yl)-1-(4-meth­oxy­phen­yl)-1*H*-phenanthro[9,10-*d*]imidazole

**DOI:** 10.1107/S1600536813003504

**Published:** 2013-02-09

**Authors:** T. Mohandas, R. Sathishkumar, P. Sakthivel, J. Jayabharathi

**Affiliations:** a Department of Physics, Shri Angalamman College of Engineering and Technology, Siruganoor, Tiruchirappalli 621 105, India; b Department of Chemistry, Annamalai University, Annamalainagar 608 002, India; c Department of Physics, Urumu Dhanalakshmi College, Tiruchirappalli 620 019, India

## Abstract

In the title compound, C_28_H_19_FN_2_O, the phenanthrene fused with an imidazole ring, constituting an essentially planar tetra­cyclic system [maximum deviation = 0.032 (2) Å], makes dihedral angles of 60.83 (4) and 80.55 (4)° with the fluoro­benzene and meth­oxy­benzene rings, respectively. The dihedral angle between the the meth­oxy­benzene and fluoro­benzene rings is 69.45 (6)°. In the crystal, C—H⋯O hydrogen bonds connect the mol­ecules into infinite strands along the *b* axis. The crystal structure is further consolidated by C—H⋯π inter­actions.

## Related literature

For background to the supra­molecular architecture of phenanthrene derivatives, see: Krebs & Spanggaard (2002 ▶); Bian *et al.* (2002 ▶); Che *et al.* (2008 ▶); Stephenson & Hardie (2006 ▶). For the crystal structures of closely related compounds, see: Yuan *et al.* (2011 ▶); Krebs *et al.* (2001 ▶).
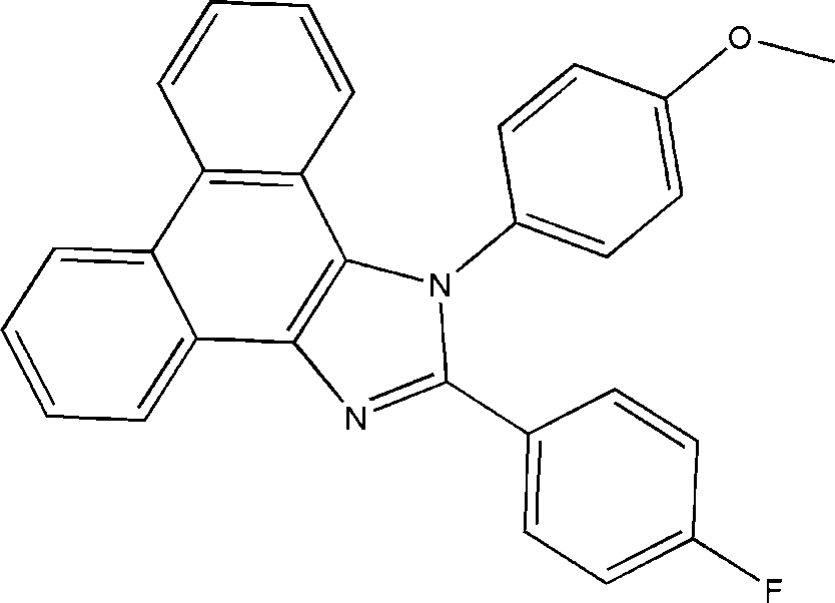


## Experimental

### 

#### Crystal data

C_28_H_19_FN_2_O
*M* *_r_* = 418.45
Triclinic, 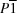

*a* = 9.6430 (3) Å
*b* = 9.8980 (3) Å
*c* = 12.3070 (4) Å
α = 77.432 (1)°
β = 71.621 (1)°
γ = 73.158 (1)°
*V* = 1056.57 (6) Å^3^
*Z* = 2
Mo *K*α radiation
μ = 0.09 mm^−1^
*T* = 293 K
0.30 × 0.20 × 0.20 mm

#### Data collection

Bruker Kappa APEXII CCD diffractometer
Absorption correction: multi-scan (*SADABS*; Bruker, 2008 ▶) *T*_min_ = 0.975, *T*_max_ = 0.983
18612 measured reflections
3723 independent reflections
3183 reflections with *I* > 2σ(*I*)
*R*_int_ = 0.026

#### Refinement

*R*[*F* ^2^ > 2σ(*F* ^2^)] = 0.035
*wR*(*F* ^2^) = 0.098
*S* = 1.03
3723 reflections
290 parameters
H-atom parameters constrained
Δρ_max_ = 0.16 e Å^−3^
Δρ_min_ = −0.22 e Å^−3^

### 

Data collection: *APEX2* (Bruker, 2008 ▶); cell refinement: *APEX2* and *SAINT* (Bruker, 2008 ▶); data reduction: *SAINT* and *XPREP* (Bruker, 2008 ▶); program(s) used to solve structure: *SHELXS97* (Sheldrick, 2008 ▶); program(s) used to refine structure: *SHELXL97* (Sheldrick, 2008 ▶); molecular graphics: *PLATON* (Spek, 2009 ▶); software used to prepare material for publication: *PLATON*.

## Supplementary Material

Crystal structure: contains datablock(s) global, I. DOI: 10.1107/S1600536813003504/pv2618sup1.cif

Structure factors: contains datablock(s) I. DOI: 10.1107/S1600536813003504/pv2618Isup2.hkl

Additional supplementary materials:  crystallographic information; 3D view; checkCIF report

## Figures and Tables

**Table 1:** Hydrogen-bond geometry (Å, °) *Cg*1, *Cg*2 and *Cg*3 are the centroids of the N1/C7/N2/C15/C16, C1–C6 and C15–C17/C22/C23/C28 rings, respectively.

*D*—H⋯*A*	*D*—H	H⋯*A*	*D*⋯*A*	*D*—H⋯*A*
C21—H21⋯O1^i^	0.93	2.59	3.426 (2)	151
C9—H9⋯*Cg*1^ii^	0.93	2.74	3.535	143
C26—H26⋯*Cg*2^iii^	0.93	2.81	3.590	143
C13—H13⋯*Cg*3^iv^	0.93	2.76	3.629	152

Symmetry codes: (i) 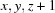; (ii) 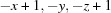; (iii) 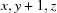; (iv) 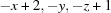.

**Fig. 1.**
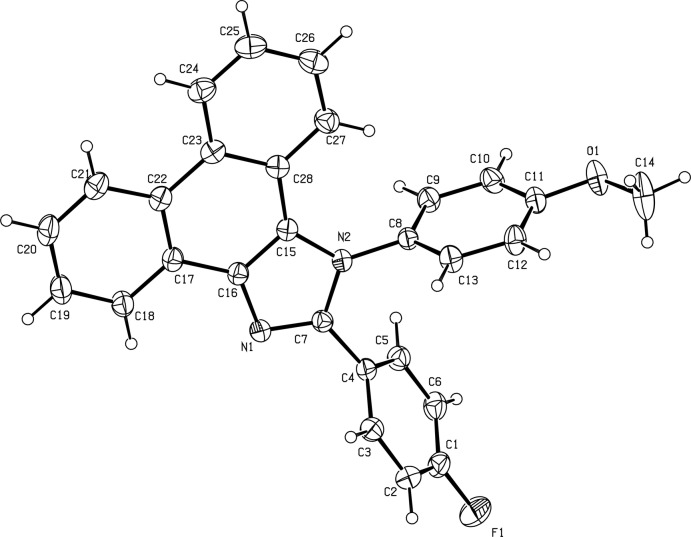
The molecular structure of the title compound with displacement ellipsoids for non-H atoms drawn at the 30% probability level.

**Fig. 2.**
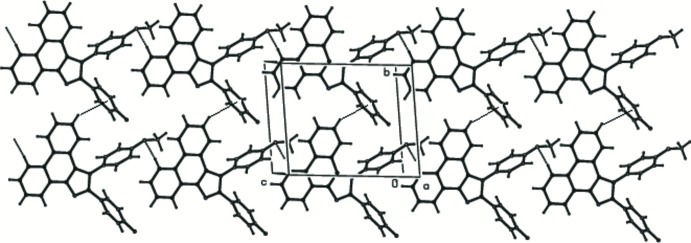
A unit cell packing diagram for the title compound. Intermolecular hydrogen bonds are shown as dashed lines.

## supplementary crystallographic information

### Comment

1*H*-phenanthro[9,10-d]imidazole can be used as a planar synthetic building block in supramolecular chemistry (Krebs & Spanggaard, 2002). Its derivatives are commonly used as ligands in metal complexes (Bian *et al.*, 2002). Some phenanthrene derivatives have excellent coordinating abilities and have been used to build supramolecular architechture (Che *et al.*, 2008; Stephenson & Hardie, 2006). We have synthesized the title compound in our laboratory and determined its crystal structure which is reported in this article.

The bond lengths and bond angles in the title compound (Fig. 1) are normal and agree very well with the corresponding bond lengths and bond angles reported for closely related compounds (Yuan *et al.*, 2011; Krebs *et al.*, 2001). The tetracyclic ring system is essentially planar (maximum deviation = 0.032 (2) Å). The dihedral angle between the fluorobenzene ring and the phenanthrene tetracyclic system is 60.83 (4)° and to that of the benzene ring of methoxybenzene is 80.55 (5)°. The dihedral angle between the methoxybenzene and the fluorobenzene rings is 69.45 (6)°. The maximum deviation of C8 atom from the mean plane of the methoxy benzene is -0.021° and that of C7 atom from that of fluorobenzene is 0.059 Å.

In the crystal packing, the molecules are linked by C—H···O intermolecular interactions into infinite chains running paralell to the *b* axis. The crystal structure is further stabilized by C—H···*Cg* interactions (Table 1), where *Cg*1 is the centre of gravity of N1/C7/N2/C15/C16, *Cg*2 is the center of gravity of C1—C6 and *Cg*3 is the center of gravity of C15/C16/C17/C22/C23/C28.

### Experimental

A mixture of phenanthrene-9,10-dione (1.0 g, 4.8 mmol), ammonium acetate (1.48 g, 19.2 mmol), 4-fluorobenzaldehyde (0.6 g, 4.3 mmol) and 4-methoxy aniline (2.95 g, 24 mmol) were refluxed in ethanol (20 ml) at 353 K. The reaction was monitored by TLC and purified by coloumn chromotography using petroleum ether:ethyl acetate (9:1) as the eluent. Yield: 0.84 g(50%). The compound was dissolved in dimethyl sulfoxide and allowed to evaporate slowly, until single crystals were grown.

### Refinement

The hydrogen atoms were placed in calculated positions with C–H = 0.93 Å to 0.96 Å, refined in the riding model with fixed isotropic displacement parameters: *U*_iso_(H) = 1.5*U*_eq_(C)for methyl group and *U*_iso_(H) = 1.2*U*_eq_(C)for other groups.

### Crystal data

**Table:**

C_28_H_19_FN_2_O	*Z* = 2
*M**_r_* = 418.45	*F*(000) = 436
Triclinic, *P*1	*D*_x_ = 1.315 Mg m^−^^3^
Hall symbol: -P 1	Mo *K*α radiation, λ = 0.71073 Å
*a* = 9.6430 (3) Å	Cell parameters from 9741 reflections
*b* = 9.8980 (3) Å	θ = 2.3–28.5°
*c* = 12.3070 (4) Å	µ = 0.09 mm^−^^1^
α = 77.432 (1)°	*T* = 293 K
β = 71.621 (1)°	Block, colourless
γ = 73.158 (1)°	0.30 × 0.20 × 0.20 mm
*V* = 1056.57 (6) Å^3^	

### Data collection

**Table:**

Bruker Kappa APEXII CCD diffractometer	3723 independent reflections
Radiation source: fine-focus sealed tube	3183 reflections with *I* > 2σ(*I*)
Graphite monochromator	*R*_int_ = 0.026
ω and φ scans	θ_max_ = 25.0°, θ_min_ = 2.2°
Absorption correction: multi-scan (*SADABS*; Bruker, 2008)	*h* = −11→11
*T*_min_ = 0.975, *T*_max_ = 0.983	*k* = −11→11
18612 measured reflections	*l* = −14→14

### Refinement

**Table:**

Refinement on *F*^2^	Secondary atom site location: difference Fourier map
Least-squares matrix: full	Hydrogen site location: inferred from neighbouring sites
*R*[*F*^2^ > 2σ(*F*^2^)] = 0.035	H-atom parameters constrained
*wR*(*F*^2^) = 0.098	*w* = 1/[σ^2^(*F*_o_^2^) + (0.0449*P*)^2^ + 0.2676*P*] where *P* = (*F*_o_^2^ + 2*F*_c_^2^)/3
*S* = 1.03	(Δ/σ)_max_ < 0.001
3723 reflections	Δρ_max_ = 0.16 e Å^−^^3^
290 parameters	Δρ_min_ = −0.22 e Å^−^^3^
0 restraints	Extinction correction: *SHELXL97*(Sheldrick, 2008), Fc^*^=kFc[1+0.001xFc^2^λ^3^/sin(2θ)]^-1/4^
Primary atom site location: structure-invariant direct methods	Extinction coefficient: 0.024 (2)

### Special details

**Table:**

Geometry. Bond distances, angles *etc*. have been calculated using the rounded fractional coordinates. All su's are estimated from the variances of the (full) variance-covariance matrix. The cell e.s.d.'s are taken into account in the estimation of distances, angles and torsion angles.
Refinement. Refinement of *F*^2^ against ALL reflections. The weighted *R*-factor *wR* and goodness of fit *S* are based on *F*^2^, conventional *R*-factors *R* are based on *F*, with *F* set to zero for negative *F*^2^. The threshold expression of *F*^2^ > σ(*F*^2^) is used only for calculating *R*-factors(gt) *etc*. and is not relevant to the choice of reflections for refinement. *R*-factors based on *F*^2^ are statistically about twice as large as those based on *F*, and *R*- factors based on ALL data will be even larger.

### Fractional atomic coordinates and isotropic or equivalent isotropic displacement parameters (Å^2^)

**Table:**

	*x*	*y*	*z*	*U*_iso_*/*U*_eq_	
F1	0.87342 (15)	−0.53172 (11)	0.19476 (9)	0.0846 (5)	
O1	0.75909 (13)	0.28562 (13)	0.05490 (9)	0.0612 (4)	
N1	0.77067 (12)	−0.21988 (11)	0.62054 (9)	0.0372 (3)	
N2	0.75022 (11)	−0.02316 (11)	0.49070 (9)	0.0347 (3)	
C1	0.8505 (2)	−0.44158 (16)	0.27061 (12)	0.0515 (5)	
C2	0.95884 (18)	−0.45772 (16)	0.32493 (13)	0.0540 (5)	
C3	0.93452 (16)	−0.36453 (15)	0.40117 (12)	0.0443 (4)	
C4	0.80356 (14)	−0.25828 (13)	0.42219 (10)	0.0352 (4)	
C5	0.69707 (16)	−0.24510 (15)	0.36438 (11)	0.0426 (4)	
C6	0.71958 (18)	−0.33777 (16)	0.28835 (12)	0.0495 (5)	
C7	0.77576 (14)	−0.16916 (13)	0.51172 (11)	0.0346 (4)	
C8	0.75523 (14)	0.05865 (13)	0.37811 (10)	0.0345 (4)	
C9	0.62372 (15)	0.12625 (15)	0.34724 (12)	0.0434 (4)	
C10	0.62899 (16)	0.20068 (15)	0.23842 (12)	0.0466 (5)	
C11	0.76621 (16)	0.20770 (15)	0.15961 (11)	0.0424 (4)	
C12	0.89759 (16)	0.13796 (17)	0.18988 (12)	0.0495 (5)	
C13	0.89114 (15)	0.06387 (15)	0.29966 (12)	0.0448 (5)	
C14	0.8964 (3)	0.2975 (3)	−0.02895 (15)	0.0935 (9)	
C15	0.72865 (13)	0.02155 (13)	0.59578 (11)	0.0345 (4)	
C16	0.74054 (14)	−0.10143 (13)	0.67333 (11)	0.0348 (4)	
C17	0.72237 (14)	−0.10002 (14)	0.79297 (11)	0.0377 (4)	
C18	0.73089 (16)	−0.22537 (16)	0.87221 (12)	0.0458 (4)	
C19	0.71638 (18)	−0.22088 (18)	0.98554 (12)	0.0542 (5)	
C20	0.69353 (18)	−0.09137 (19)	1.02226 (13)	0.0567 (5)	
C21	0.68346 (17)	0.03217 (18)	0.94647 (12)	0.0505 (5)	
C22	0.69645 (14)	0.03262 (15)	0.82916 (11)	0.0395 (4)	
C23	0.68514 (14)	0.16372 (14)	0.74603 (12)	0.0400 (4)	
C24	0.65660 (17)	0.29785 (16)	0.78018 (14)	0.0515 (5)	
C25	0.64649 (19)	0.42041 (17)	0.70328 (15)	0.0582 (6)	
C26	0.66392 (18)	0.41572 (16)	0.58804 (15)	0.0558 (6)	
C27	0.69159 (16)	0.28783 (15)	0.55041 (13)	0.0468 (5)	
C28	0.70222 (14)	0.15991 (14)	0.62762 (11)	0.0376 (4)	
H2	1.04664	−0.52960	0.31090	0.0648*	
H3	1.00705	−0.37321	0.43891	0.0532*	
H5	0.60945	−0.17285	0.37710	0.0511*	
H6	0.64780	−0.33001	0.25013	0.0594*	
H9	0.53134	0.12146	0.40014	0.0520*	
H10	0.54020	0.24635	0.21777	0.0559*	
H12	0.99020	0.14075	0.13664	0.0594*	
H13	0.97971	0.01726	0.32035	0.0538*	
H14A	0.87564	0.35433	−0.09847	0.1400*	
H14B	0.95092	0.34195	0.00023	0.1400*	
H14C	0.95552	0.20428	−0.04544	0.1400*	
H18	0.74654	−0.31228	0.84751	0.0550*	
H19	0.72181	−0.30439	1.03784	0.0651*	
H20	0.68497	−0.08855	1.09919	0.0680*	
H21	0.66766	0.11797	0.97296	0.0606*	
H24	0.64426	0.30339	0.85727	0.0618*	
H25	0.62775	0.50759	0.72854	0.0698*	
H26	0.65681	0.49957	0.53619	0.0670*	
H27	0.70353	0.28537	0.47281	0.0562*	

### Atomic displacement parameters (Å^2^)

**Table:**

	*U*^11^	*U*^22^	*U*^33^	*U*^12^	*U*^13^	*U*^23^
F1	0.1259 (10)	0.0703 (7)	0.0668 (7)	−0.0203 (7)	−0.0242 (6)	−0.0351 (5)
O1	0.0734 (8)	0.0768 (8)	0.0390 (6)	−0.0346 (6)	−0.0239 (5)	0.0159 (5)
N1	0.0405 (6)	0.0369 (6)	0.0335 (6)	−0.0077 (5)	−0.0115 (5)	−0.0032 (4)
N2	0.0374 (6)	0.0361 (6)	0.0299 (5)	−0.0095 (4)	−0.0095 (4)	−0.0018 (4)
C1	0.0762 (11)	0.0449 (8)	0.0351 (7)	−0.0191 (8)	−0.0098 (7)	−0.0105 (6)
C2	0.0594 (9)	0.0454 (8)	0.0468 (8)	0.0009 (7)	−0.0089 (7)	−0.0113 (7)
C3	0.0436 (8)	0.0456 (8)	0.0415 (7)	−0.0048 (6)	−0.0138 (6)	−0.0060 (6)
C4	0.0385 (7)	0.0354 (7)	0.0303 (6)	−0.0103 (5)	−0.0082 (5)	−0.0012 (5)
C5	0.0418 (7)	0.0463 (8)	0.0395 (7)	−0.0085 (6)	−0.0128 (6)	−0.0055 (6)
C6	0.0603 (9)	0.0570 (9)	0.0387 (7)	−0.0216 (8)	−0.0182 (7)	−0.0045 (7)
C7	0.0326 (6)	0.0360 (7)	0.0339 (7)	−0.0071 (5)	−0.0095 (5)	−0.0032 (5)
C8	0.0375 (7)	0.0349 (7)	0.0307 (6)	−0.0102 (5)	−0.0087 (5)	−0.0023 (5)
C9	0.0343 (7)	0.0494 (8)	0.0397 (7)	−0.0076 (6)	−0.0068 (6)	−0.0006 (6)
C10	0.0440 (8)	0.0481 (8)	0.0467 (8)	−0.0072 (6)	−0.0201 (6)	0.0020 (6)
C11	0.0546 (8)	0.0445 (8)	0.0333 (7)	−0.0209 (6)	−0.0156 (6)	0.0017 (6)
C12	0.0419 (8)	0.0664 (10)	0.0369 (7)	−0.0213 (7)	−0.0046 (6)	0.0022 (7)
C13	0.0354 (7)	0.0552 (9)	0.0420 (8)	−0.0121 (6)	−0.0121 (6)	0.0013 (6)
C14	0.0953 (15)	0.150 (2)	0.0433 (10)	−0.0706 (15)	−0.0220 (10)	0.0309 (11)
C15	0.0314 (6)	0.0392 (7)	0.0331 (7)	−0.0088 (5)	−0.0088 (5)	−0.0047 (5)
C16	0.0326 (6)	0.0390 (7)	0.0330 (7)	−0.0086 (5)	−0.0095 (5)	−0.0044 (5)
C17	0.0331 (7)	0.0463 (7)	0.0333 (7)	−0.0092 (6)	−0.0094 (5)	−0.0045 (6)
C18	0.0495 (8)	0.0493 (8)	0.0370 (7)	−0.0104 (6)	−0.0136 (6)	−0.0018 (6)
C19	0.0598 (9)	0.0637 (10)	0.0354 (8)	−0.0143 (8)	−0.0147 (7)	0.0031 (7)
C20	0.0605 (10)	0.0765 (11)	0.0333 (7)	−0.0145 (8)	−0.0139 (7)	−0.0086 (7)
C21	0.0499 (8)	0.0625 (9)	0.0419 (8)	−0.0117 (7)	−0.0119 (7)	−0.0162 (7)
C22	0.0324 (7)	0.0495 (8)	0.0376 (7)	−0.0090 (6)	−0.0095 (5)	−0.0092 (6)
C23	0.0324 (7)	0.0450 (8)	0.0452 (8)	−0.0089 (6)	−0.0112 (6)	−0.0112 (6)
C24	0.0526 (9)	0.0509 (9)	0.0567 (9)	−0.0097 (7)	−0.0176 (7)	−0.0184 (7)
C25	0.0623 (10)	0.0434 (8)	0.0745 (11)	−0.0118 (7)	−0.0198 (8)	−0.0190 (8)
C26	0.0601 (10)	0.0392 (8)	0.0688 (11)	−0.0164 (7)	−0.0181 (8)	−0.0017 (7)
C27	0.0513 (8)	0.0422 (8)	0.0478 (8)	−0.0154 (6)	−0.0133 (7)	−0.0023 (6)
C28	0.0325 (7)	0.0395 (7)	0.0418 (7)	−0.0100 (5)	−0.0101 (5)	−0.0059 (6)

### Geometric parameters (Å, º)

**Table:**

F1—C1	1.3544 (19)	C20—C21	1.366 (2)
O1—C11	1.3612 (17)	C21—C22	1.4084 (19)
O1—C14	1.417 (3)	C22—C23	1.4673 (19)
N1—C7	1.3146 (17)	C23—C24	1.405 (2)
N1—C16	1.3770 (16)	C23—C28	1.4217 (19)
N2—C7	1.3737 (16)	C24—C25	1.366 (2)
N2—C8	1.4388 (16)	C25—C26	1.384 (2)
N2—C15	1.3920 (17)	C26—C27	1.367 (2)
C1—C2	1.364 (3)	C27—C28	1.407 (2)
C1—C6	1.367 (2)	C2—H2	0.9300
C2—C3	1.378 (2)	C3—H3	0.9300
C3—C4	1.382 (2)	C5—H5	0.9300
C4—C5	1.386 (2)	C6—H6	0.9300
C4—C7	1.4710 (18)	C9—H9	0.9300
C5—C6	1.376 (2)	C10—H10	0.9300
C8—C9	1.379 (2)	C12—H12	0.9300
C8—C13	1.368 (2)	C13—H13	0.9300
C9—C10	1.374 (2)	C14—H14A	0.9600
C10—C11	1.382 (2)	C14—H14B	0.9600
C11—C12	1.378 (2)	C14—H14C	0.9600
C12—C13	1.382 (2)	C18—H18	0.9300
C15—C16	1.3742 (18)	C19—H19	0.9300
C15—C28	1.4361 (18)	C20—H20	0.9300
C16—C17	1.4296 (18)	C21—H21	0.9300
C17—C18	1.401 (2)	C24—H24	0.9300
C17—C22	1.407 (2)	C25—H25	0.9300
C18—C19	1.367 (2)	C26—H26	0.9300
C19—C20	1.386 (2)	C27—H27	0.9300
			
C11—O1—C14	117.86 (15)	C24—C23—C28	117.36 (13)
C7—N1—C16	104.70 (10)	C23—C24—C25	121.83 (15)
C7—N2—C8	123.87 (10)	C24—C25—C26	120.46 (15)
C7—N2—C15	106.27 (10)	C25—C26—C27	120.02 (15)
C8—N2—C15	129.77 (11)	C26—C27—C28	120.82 (14)
F1—C1—C2	118.77 (15)	C15—C28—C23	116.05 (12)
F1—C1—C6	118.08 (16)	C15—C28—C27	124.42 (12)
C2—C1—C6	123.15 (15)	C23—C28—C27	119.53 (12)
C1—C2—C3	118.13 (15)	C1—C2—H2	121.00
C2—C3—C4	120.75 (15)	C3—C2—H2	121.00
C3—C4—C5	119.16 (12)	C2—C3—H3	120.00
C3—C4—C7	119.08 (13)	C4—C3—H3	120.00
C5—C4—C7	121.60 (12)	C4—C5—H5	120.00
C4—C5—C6	120.71 (14)	C6—C5—H5	120.00
C1—C6—C5	118.09 (16)	C1—C6—H6	121.00
N1—C7—N2	112.64 (11)	C5—C6—H6	121.00
N1—C7—C4	123.91 (11)	C8—C9—H9	120.00
N2—C7—C4	123.44 (11)	C10—C9—H9	120.00
N2—C8—C9	120.21 (12)	C9—C10—H10	120.00
N2—C8—C13	119.79 (12)	C11—C10—H10	120.00
C9—C8—C13	119.93 (12)	C11—C12—H12	120.00
C8—C9—C10	120.09 (14)	C13—C12—H12	120.00
C9—C10—C11	120.04 (15)	C8—C13—H13	120.00
O1—C11—C10	115.43 (14)	C12—C13—H13	120.00
O1—C11—C12	124.75 (14)	O1—C14—H14A	109.00
C10—C11—C12	119.82 (13)	O1—C14—H14B	109.00
C11—C12—C13	119.72 (14)	O1—C14—H14C	109.00
C8—C13—C12	120.40 (14)	H14A—C14—H14B	109.00
N2—C15—C16	104.95 (11)	H14A—C14—H14C	109.00
N2—C15—C28	132.25 (11)	H14B—C14—H14C	110.00
C16—C15—C28	122.79 (12)	C17—C18—H18	120.00
N1—C16—C15	111.44 (11)	C19—C18—H18	120.00
N1—C16—C17	126.47 (11)	C18—C19—H19	120.00
C15—C16—C17	122.10 (12)	C20—C19—H19	120.00
C16—C17—C18	121.87 (12)	C19—C20—H20	120.00
C16—C17—C22	117.60 (12)	C21—C20—H20	120.00
C18—C17—C22	120.53 (12)	C20—C21—H21	119.00
C17—C18—C19	120.48 (14)	C22—C21—H21	119.00
C18—C19—C20	119.75 (15)	C23—C24—H24	119.00
C19—C20—C21	120.61 (14)	C25—C24—H24	119.00
C20—C21—C22	121.54 (15)	C24—C25—H25	120.00
C17—C22—C21	117.08 (13)	C26—C25—H25	120.00
C17—C22—C23	120.21 (12)	C25—C26—H26	120.00
C21—C22—C23	122.71 (13)	C27—C26—H26	120.00
C22—C23—C24	121.43 (13)	C26—C27—H27	120.00
C22—C23—C28	121.21 (12)	C28—C27—H27	120.00
			
C14—O1—C11—C10	179.46 (17)	O1—C11—C12—C13	178.88 (14)
C14—O1—C11—C12	−0.7 (2)	C11—C12—C13—C8	0.4 (2)
C7—N1—C16—C15	−0.54 (16)	N2—C15—C16—N1	0.91 (16)
C7—N1—C16—C17	179.29 (14)	C28—C15—C16—C17	2.1 (2)
C16—N1—C7—N2	−0.08 (17)	N2—C15—C28—C23	179.92 (15)
C16—N1—C7—C4	−179.26 (14)	N2—C15—C28—C27	0.4 (3)
C8—N2—C15—C16	−177.56 (13)	C16—C15—C28—C23	−1.5 (2)
C15—N2—C7—C4	179.82 (13)	C16—C15—C28—C27	179.03 (15)
C7—N2—C15—C16	−0.91 (15)	N2—C15—C16—C17	−178.92 (13)
C8—N2—C7—N1	177.54 (13)	C28—C15—C16—N1	−178.04 (13)
C7—N2—C8—C9	100.31 (16)	N1—C16—C17—C18	−1.4 (2)
C15—N2—C7—N1	0.64 (16)	N1—C16—C17—C22	178.10 (14)
C8—N2—C7—C4	−3.3 (2)	C15—C16—C17—C18	178.38 (15)
C15—N2—C8—C13	99.46 (17)	C15—C16—C17—C22	−2.1 (2)
C7—N2—C15—C28	177.90 (15)	C16—C17—C18—C19	178.53 (15)
C8—N2—C15—C28	1.3 (2)	C22—C17—C18—C19	−1.0 (2)
C7—N2—C8—C13	−76.67 (18)	C16—C17—C22—C21	−178.05 (14)
C15—N2—C8—C9	−83.56 (18)	C16—C17—C22—C23	1.5 (2)
F1—C1—C2—C3	179.49 (14)	C18—C17—C22—C21	1.5 (2)
F1—C1—C6—C5	−179.24 (13)	C18—C17—C22—C23	−178.97 (14)
C6—C1—C2—C3	−0.1 (2)	C17—C18—C19—C20	−0.2 (3)
C2—C1—C6—C5	0.3 (2)	C18—C19—C20—C21	0.8 (3)
C1—C2—C3—C4	0.3 (2)	C19—C20—C21—C22	−0.3 (3)
C2—C3—C4—C7	174.59 (13)	C20—C21—C22—C17	−0.9 (2)
C2—C3—C4—C5	−0.8 (2)	C20—C21—C22—C23	179.60 (16)
C3—C4—C7—N2	123.12 (15)	C17—C22—C23—C24	178.83 (15)
C5—C4—C7—N1	117.51 (16)	C17—C22—C23—C28	−1.0 (2)
C3—C4—C7—N1	−57.8 (2)	C21—C22—C23—C24	−1.7 (2)
C7—C4—C5—C6	−174.21 (13)	C21—C22—C23—C28	178.55 (15)
C5—C4—C7—N2	−61.58 (19)	C22—C23—C24—C25	179.73 (16)
C3—C4—C5—C6	1.1 (2)	C28—C23—C24—C25	−0.5 (2)
C4—C5—C6—C1	−0.8 (2)	C22—C23—C28—C15	0.9 (2)
C9—C8—C13—C12	0.8 (2)	C22—C23—C28—C27	−179.59 (14)
N2—C8—C13—C12	177.75 (13)	C24—C23—C28—C15	−178.94 (14)
C13—C8—C9—C10	−1.0 (2)	C24—C23—C28—C27	0.6 (2)
N2—C8—C9—C10	−178.00 (12)	C23—C24—C25—C26	0.2 (3)
C8—C9—C10—C11	0.1 (2)	C24—C25—C26—C27	−0.1 (3)
C9—C10—C11—C12	1.1 (2)	C25—C26—C27—C28	0.2 (3)
C9—C10—C11—O1	−179.12 (13)	C26—C27—C28—C15	179.01 (16)
C10—C11—C12—C13	−1.3 (2)	C26—C27—C28—C23	−0.5 (2)

### Hydrogen-bond geometry (Å, º)

Cg1, Cg2 and Cg3 are the centroids of the N1/C7/N2/C15/C16, C1–C6 and C15–C17/C22/C23/C28 rings, respectively.

**Table:**

*D*—H···*A*	*D*—H	H···*A*	*D*···*A*	*D*—H···*A*
C21—H21···O1^i^	0.93	2.59	3.426 (2)	151
C9—H9···*Cg*1^ii^	0.93	2.74	3.535	143
C26—H26···*Cg*2^iii^	0.93	2.81	3.590	143
C13—H13···*Cg*3^iv^	0.93	2.76	3.629	152

Symmetry codes: (i) *x*, *y*, *z*+1; (ii) −*x*+1, −*y*, −*z*+1; (iii) *x*, *y*+1, *z*; (iv) −*x*+2, −*y*, −*z*+1.
